# Correction: Han et al. Genotypic Homogeneity in Distinctive Transforming Growth Factor-Beta-Induced (TGFBI) Protein Phenotypes. *Int. J. Mol. Sci.* 2021, *22*, 1230

**DOI:** 10.3390/ijms26052273

**Published:** 2025-03-04

**Authors:** Sang Beom Han, Venkatraman Anandalakshmi, Chee Wai Wong, Si Rui Ng, Jodhbir S. Mehta

**Affiliations:** 1 Department of Ophthalmology, Kangwon National University School of Medicine, Chuncheon 24289, Republic of Korea; msbhan@nate.com; 2 Department of Ophthalmology, Kangwon National University Hospital, Chuncheon 24289, Republic of Korea; 3 Singapore Eye Research Institute, Singapore 169856, Singapore; vidhyavraman@yahoo.com (V.A.); wongcheewai81@gmail.com (C.W.W.); sirui.ng@mohh.com.sg (S.R.N.); 4 Singapore National Eye Centre, Singapore 168751, Singapore; 5 Ophthalmology and Visual Sciences Academic Clinical Program, Duke-NUS Graduate Medical School, Singapore 169857, Singapore

## Text Correction

There was an error in the original publication [1]. In the following sentence, ‘H626P’ in the cited papers was written as ‘H626R’. “However, H626R mutation was also reported to be able to cause a significantly different phenotype that was clinically similar to TBCD and RBCD [36,37], which also suggests that the same mutations can result in various phenotypes [5].”

A correction has been made to the Discussion, fourth paragraph.

“A further mutation at the same base pair resulting in H626P has also been described in LCD as well as in significantly different phenotypes that were clinically similar to TBCD and RBCD [36,37], suggesting that mutations at this site can result in various phenotypes [5].”

The authors state that the scientific conclusions are unaffected. This correction was approved by the Academic Editor. The original publication has also been updated.
